# Acute effects of insulin and insulin‐induced hypoglycaemia on carotid body chemoreceptor activity and cardiorespiratory responses in dogs

**DOI:** 10.1113/EP090584

**Published:** 2022-12-02

**Authors:** Santhosh M. Baby, Faisal Zaidi, Gerald E. Hunsberger, David Sokal, Isha Gupta, Silvia V. Conde, Daniel Chew, Kristen Rall, Robert W. Coatney

**Affiliations:** ^1^ Translational Sciences and Treatment Discovery Galvani Bioelectronics Collegeville PA USA; ^2^ Research Devices and Technology Galvani Bioelectronics Collegeville PA USA; ^3^ Experimental Medicine Surgical Development and Therapy Galvani Bioelectronics Stevenage UK; ^4^ NOVA Medical School Faculdade de Ciências Médicas Universidade Nova de Lisboa Lisboa Portugal

**Keywords:** carotid body, carotid body chemoreceptors, dogs, hypoglycaemia, insulin, sodium cyanide

## Abstract

The carotid body chemoreceptors (CBC) play an important role in the adaptive counter‐regulatory response to hypoglycaemia by evoking the CBC‐mediated sympathetic neuronal system to restore normoglycaemia. *Ex vivo* studies have shown varied responses of insulin‐induced hypoglycaemia on CBC function, and several in vivo studies have indirectly established the role of CBCs in restoring normoglycaemia in both animals and humans. However, a direct effect of insulin and/or insulin‐induced hypoglycaemia on CBC activity is not established in animal models. Therefore, the aim of this study was to evaluate in vivo effects of insulin and insulin‐induced hypoglycaemia on CBC activity and cardiorespiration in a preclinical large animal model. The carotid sinus nerve (CSN) activity and cardiorespiratory responses to sodium cyanide (NaCN; 25 µg/kg) were compared before (euglycaemic) and after (hypoglycaemic) intracarotid administration of insulin (12.5–100 µU/dogs) in beagle dogs. Insulin administration increased CSN activity and minute ventilation ($\dot{V}$_E_) with significant (*P* < 0.0001) effects on heart rate and blood pressure. Insulin‐mediated effects on CSN and cardiorespiration were sustained and the change in $\dot{V}$_E_ was driven by tidal volume only. Insulin significantly (*P <* 0.0001) lowered blood glucose level. NaCN‐mediated CSN activity and $\dot{V}$_E_ were significantly (*P* < 0.0001) augmented during insulin‐induced hypoglycaemia. The augmented $\dot{V}$_E_ was primarily driven by respiratory frequency and partially by tidal volume. The cardiovascular reflex response mediated through CBC stimulation was significantly (*P* < 0.0001) exacerbated during insulin‐induced hypoglycaemia. Collectively, these results demonstrate direct effects of insulin and insulin‐induced hypoglycaemia on CBC chemosensitivity to potentiate CBC‐mediated neuroregulatory pathways to initiate adaptive neuroendocrine and cardiorespiratory counter‐regulatory responses to restore normoglycaemia.

## Abstract (highlights)

- **What is the central question of this study?**
  What are the effects of insulin and insulin‐induced hypoglycaemia on carotid body chemoreceptor activity in vivo and how do carotid body chemoreceptor stimulation‐mediated cardiorespiratory responses in beagle dogs compare during euglycaemia and insulin‐induced hypoglycaemia?
- **What is the main finding and its importance?**
  Intracarotid insulin administration leads to sustained increase in carotid body chemoreceptor activity and respiratory response with significant cardiovascular effects. Insulin‐induced hypoglycaemia exacerbated NaCN‐mediated carotid body chemoreceptor activity and respiratory response with enhanced cardiovascular reflex response. These findings suggest that insulin‐induced hypoglycaemia augments the carotid body chemoreceptors to initiate the adaptive counter‐regulatory responses to restore the normoglycaemic condition.

## 1 INTRODUCTION

The brain primarily relies upon glucose for metabolism. Thus, any variation in blood glucose level triggers highly integrated rapid neuroendocrine counter‐regulatory mechanisms to increase or decrease glucose production and/or utilization for maintaining the normal metabolic status. Although there are multiple central and peripheral glucose sensors in locations including the hypothalamus, hindbrain, gastrointestinal tract, portal mesenteric vein, pancreas and liver (Bohland et al., 2014), their relative contribution to the counter‐regulatory response to hypoglycaemia is not completely understood.

Accumulating evidence suggests that apart from its well‐established role in sensing arterial blood gases and pH (Kumar & Prabhakar, 2012; Lahiri et al., 2006; Lopez‐Barneo, 2018), the carotid body chemoreceptors (CBC) may also play a significant role in mediating counter‐regulatory responses to hypoglycaemia in animals and humans (Conde et al., 2018; Holmes et al., 2019; Joyner et al., 2018). The exact role and the molecular mechanisms by which the CBCs responds to changes in arterial blood glucose is not fully understood. However, bilateral transection of carotid sinus nerve (CSNx) or desensitization of the CBCs with hyperoxia completely and/or partially abolished hypoglycaemia‐induced counter‐regulatory responses in animals (Bin‐Jaliah et al., 2004; Koyama et al., 2000; Thompson et al., 2016) and humans (Wehrwein et al., 2015), suggesting a significant role of the CBCs in hypoglycaemia‐induced counter‐regulatory responses.

*Ex vivo* studies in rodents have produced conflicting findings regarding the role of the CBCs in hypoglycaemia‐induced counter‐regulatory responses. Studies using carotid body glomus primary cell culture and/or carotid body slice preparations have shown that low glucose concentrations induced an acute neurosecretory response in glomus cells through inhibition of K^+^ currents (Garcia‐Fernandez et al., 2007; Pardal & Lopez‐Barneo, 2002; Zhang et al., 2007). Conversely, in freshly isolated, intact carotid bodies and glomus cells, this acute neurosecretory response to low glucose levels was absent (Bin‐Jaliah et al., 2004; Conde et al., 2007; Gallego‐Martin et al., 2012; Holmes et al., 2014). These discrepancies could result from differences in carotid body sample preparation and/or limitations in the experimental designs. Regardless of these discrepancies, available experimental data suggest that directly and/or indirectly the CBCs play a major role in glucose sensing in mammals and trigger an adaptive counter‐regulatory response that has potential pathophysiological implications. in vivo studies have shown that CBC activity is modified by metabolic factors that contribute to glucose homeostasis. CBC overactivity was reported with insulin resistance and arterial hypertension in animal models of the metabolic syndrome and prediabetes (Cracchiolo et al., 2019; Ribeiro et al., 2013) and in prediabetic patients (Cunha‐Guimaraes et al., 2020), and CSNx prevents and reverts insulin resistance and arterial hypertension induced by high‐energy diets (Ribeiro et al., 2013; Sacramento et al., 2018). In addition to functional abolition of CBC activity by bilateral CSNx, bioelectronic suppression of CSN hyperactivity in high‐energy diets improved metabolic control in rat models of type 2 diabetes (Sacramento et al., 2018) suggesting the translational potential of bioelectronic therapy for treating metabolic diseases in humans. Several studies have shown that CBC activity is modified by metabolic factors that contribute to glucose homeostasis, such as insulin and leptin (Caballero‐Eraso et al., 2019; Ribeiro et al., 2013; Ribeiro et al., 2018), and disruption of insulin signaling might contribute to dysmetabolic states (Conde et al., 2018). However, till today there are no clear in vivo data supporting the direct action of insulin on CBC output. Therefore, we used an in vivo canine model to record CSN action potentials and cardiorespiratory activities to understand the direct effects of insulin and insulin‐induced hypoglycaemia on CBC activity and cardiorespiratory responses using a known peripheral chemoreceptor stimulant, sodium cyanide (NaCN), to explore the translational potential of novel bioelectronic therapy in a preclinical large animal model. Acute administration of insulin and/or insulin‐induced hypoglycaemia leads to sustained increase in CSN activity with comparable changes in respiratory and cardiovascular parameters. Further, insulin‐induced hypoglycaemia increased the resting CSN activity, ventilation and augmented NaCN‐mediated ventilatory responses with a corresponding increase in peripheral chemoreceptor activity in anaesthetized beagle dogs.

## 2 METHODS

### 2.1 Ethical approval

All animals used in this study were cared for and used humanely in accordance with the requirements specified by the Galvani Bioelectronics Policy on the Care, Welfare and Treatment of Animals and in accordance with the *Guide for the Care and Use of Laboratory Animals* (National Research Council NIH publications No. 85‐23, revised 1996). Adult male beagle dogs procured from Marshall Farms (*n* = 9, body weight mean ± SD: 11.7 ± 1.0 kg; Marshall BioResources, North Rose, NY, USA) were fed certified canine diet with nutrients, minerals and vitamins (LabDiet, St Louis, MO, USA). The dogs were housed at the GlaxoSmithKline King of Prussia (PA, USA) facility, which met American Association for the Accreditation of Laboratory Animal Care (AAALAC) guidelines, and the protocols were approved by the GSK‐Galvani Institutional Animal Care and Use Committee (IACUC; Protocol GAL‐3012).

### 2.2 Anaesthesia and surgery

Prior to surgery, male adult beagle dogs were fasted overnight with free access to drinking water. Dogs were initially pre‐medicated with diazepam (0.25 mg/kg: Hospira, Inc., Lake Forest, IL, USA) anaesthetized with ketamine (2.5 mg/kg; Vedco, Inc., St Joseph, MO, USA) and dexdomitor (0.025 mg/kg; Zoetis, Kalamazoo, MI, USA) using an indwelling cephalic catheter. The optimum level of anaesthesia was maintained by continuous infusion of the anaesthetics. The depth of anaesthesia was assessed periodically by testing the palpebral responses and heart rate. Body temperature was maintained at 37°C using a homoeothermic water‐blanket (Medi‐Therm III, Gaymar Industries, Inc., Orchard Park, NY, USA). Dogs were intubated with endotracheal tube (ID: 7–8 mm; Protex Bivona, Smith Medicals, Dublin, OH, USA) and transferred to the operating room. During surgical procedures, animals were mechanically ventilated (Drager Narkomed GS, Auxo Medical LLC, Richmond, VA, USA) on medical grade air with a supplement of oxygen to keep the arterial oxygen saturation close to 100% to keep the carotid body quiescent. For delivery of vehicle (saline), NaCN (25 µg/kg) and insulin (12.5–100 µU/dog) close to the CBC, both common carotid arteries were cannulated with heparin‐coated catheter (3.5 Fr; Instech Solomon, Plymouth Meeting, PA, USA) at least 4–5 cm distal to the bifurcation of common carotid artery into internal and external carotid arteries. The tip of the catheter was placed ∼2 cm below the bifurcation to prevent potential backflow of injectate into the aortic arch.

### 2.3 Cardiovascular measurement

The electrocardiogram (ECG) bio‐potentials were amplified (BioAmp FE132, ADInstruments, Inc., Colorado Springs, CO, USA), digitized (PowerLab, ADInstruments) and continuously recorded at 1000/s rate (LabChart‐8 Pro, ADInstruments). The cyclic measurement function in LabChart was used for calculating heart rate (HR) from the ECG signals by adjusting the standard deviation of the peak height to mark all QRS complexes. The right femoral artery was cannulated using silicone catheters (Access Technologies, Skokie, IL, USA) and connected to a fluid‐filled pressure transducer to enable continuous monitoring of arterial blood pressure (Deltran fluid filled pressure transducer, Utah Medical Products, Inc., Midvale, UT, USA). The pressure signal was amplified using bridge amplifier (FE221, ADInstruments), digitized (PowerLab) and continuously recorded at 1000/s (LabChart‐8 Pro). The cyclic average minimum (diastolic blood pressure; DBP), the cyclic average maximum (systolic blood pressure; SBP) and the weighted average of 1/3 max + 2/3 min (mean arterial blood pressure; MAP) algorithm of the blood pressure module in the LabChart was used for calculating DBP, SBP and MAP, respectively. Arterial blood gases and pH were periodically monitored using an I‐Stat machine (Abaxis, Inc., Union City, CA, USA). The femoral venous vessel was used for intravenous administration of fluids and sampling venous blood for periodic glucose monitoring (Quintet AC, McKesson Medical Surgical, Inc., Richmond, VA, USA).

### 2.4 Respiratory measurement

Following surgical procedures, animals were weaned gradually from the mechanical ventilator by increasing the respiratory drive and stabilized for 45–60 min to recover from the surgical stress. The endotracheal tube (7–8 mm ID, Bivona, Gary, IN, USA) was connected to the pneumotachometer (MLT 10L, ADInstruments) and differential pressure transducer (FE141, ADInstruments) to measure respiratory airflow. The airflow signal was filtered (low pass 100 Hz), and the cyclic measurement function in LabChart was used to calculate respiratory frequency (*f*_R_ – in breaths/min). Tidal volume (*V*_T_) was calculated by integrating inspiratory deflection of the airflow waveform using the integral function within LabChart. Minute ventilation ($\dot{V}$_E_) was calculated as the product of *f*_R_ and *V*_T_. A pneumotachometer and Deltran fluid filled pressure transducer were calibrated before each experiment. Respiratory flow was digitized and recorded at 1000/s (PowerLab), continuously recorded (LabChart‐8 Pro) and stored for data analysis. Volume calibrations was performed before each experiment with a 2.5 litres/s volume using a 10‐litre syringe (ADInstruments).

### 2.5 Carotid body chemoreceptor activity

Carotid sinus nerve (CSN) on both sides was exposed and cleared where it branches off from the glossopharyngeal nerve to record the afferent nerve activity. Compound action potentials from both CSNs were recorded using cuff electrodes (inner diameter: 800–1500 µm, CorTec GmbH, Frieburg, Germany). The cuff electrodes were secured to the nerves and lead wires from electrodes were exteriorized and connected to high‐impedance head stages (HS‐1, CWE, Inc., Ardmore, PA, USA). The CSN signals were amplified (BMA‐400, CWE, Inc.), filtered (digital filter high‐pass 100 Hz and low‐pass 1000 Hz with mains filter and AC coupling), digitized at 1000–10,000/s (PowerLab) and continuously recorded (LabChart). The integrated CSN signals were generated from the raw CSN neurogram using the integral function in LabChart at constant time decay and stored for data analysis.

### 2.6 in vivo experimental protocol

The ECG, respiratory flow, blood pressure and CSN activities were recorded continuously during the experiment. Arterial blood samples were periodically collected for arterial blood gases and pH measurement. Similarly, blood glucose level was monitored periodically and maintained at euglycaemic level (120–130 mg/dl) by adjusting the dextrose infusion rate (0.5 g/ml Hospira, Inc.). All solutions for intracarotid administration were kept at 35–37°C prior to injection. After a stable baseline recording during the euglycaemic state, 50 µl of saline and 200 µl of saline (flush) at 37°C were delivered into the left common carotid artery, and the CSN responses and the cardiorespiratory responses were recorded for 30 min. This was followed by saline administration and NaCN (Sigma‐Aldrich, St Louis, MO, USA) at 25 µg/kg in 50 µl of saline, and an additional 200 µl saline (flush) at 37°C was delivered at the left common carotid artery followed by recording for 30 min. Saline and NaCN‐mediated responses were repeated at the right common carotid artery. In six dogs, after finishing saline and NaCN challenges during euglycaemia, hypoglycaemia was achieved by intracarotid bolus administration of insulin (100, 50, 25 and 12.5 U/dog, single dose; Eli Lilly, Indianapolis, IN, USA). If required, dextrose infusion rate was adjusted to keep the desired blood glucose level (45–50 mg/dl). Insulin‐mediated changes in CSN activity, respiratory flow and arterial blood pressure waveform were continuously recorded. After a stable blood glucose level (45–50 mg/dl) during insulin‐induced hypoglycaemia, intra‐carotid administration of saline and NaCN was repeated. During euglycaemia, saline and NaCN‐mediated responses were compared in all nine (*n* = 9) animals and during insulin‐induced hypoglycaemia saline and NaCN‐mediated responses were compared only in six animals (*n* = 6).

### 2.7 Killing

Experiments were terminated, and dogs were killed with an overdose (5 ml/animal) of pentobarbital sodium (360 mg/ml; Fatal‐Plus, Votech Pharmaceuticals, Ltd, Dearborn, MI, USA). Death was confirmed by auscultation. In addition, cessation of physiological parameters, respiratory flow, blood pressure waveform and ECG signals were monitored to confirm death.

### 2.8 Statistical analysis

All data are presented as the mean ± SD. GraphPad Prism (version 8.3.0) software (GraphPad Software, Inc., San Diego, CA, USA) was used for data analysis and generating graphs. The CSN activity, cardiovascular (HR, DBP, SBP and MAP) and respiratory (*f*_R_, *V*_T_ and ${\dot{V}}_{E}$) data were expressed as the percentage change from the preceding baseline above the baseline. In six dogs, the effects of insulin and NaCN on ventilation, HR, blood pressure and CSN activity during euglycaemia and insulin‐induced hypoglycaemia were evaluated using two‐way ANOVA with repeated measure on treatment and time. When differences were detected with ANOVA, multiple comparisons tests were used to detect differences between means followed by Šidák's *post hoc* test. Differences in baseline, cardiovascular (HR, DBP, SBP and MAP), respiratory parameters (*f*_R_, *V*_T_ and ${\dot{V}}_{E}$) and arterial blood gases and pH during euglycaemia and insulin‐induced hypoglycaemia were compared using Student's unpaired *t* test with Welch's correction. Differences between means were considered significant when *P* ≤ 0.05.

## 3 RESULTS

### 3.1 Effect of NaCN on carotid sinus nerve activity and cardiorespiratory parameters

**FIGURE 1:**
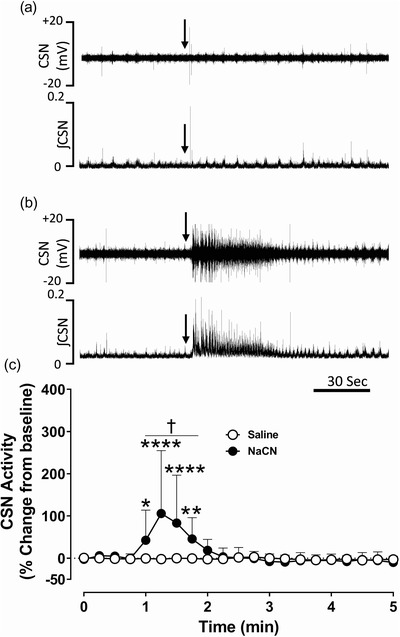
Sodium cyanide (NaCN) transiently increased carotid sinus nerve (CSN) activity during euglycaemia in beagle dogs. (a, b) Representative recordings of in vivo CSN activity following intracarotid administration of saline (a) and NaCN (25 µg/kg) (b) during euglycaemia. Upper panel: CSN raw signal; and lower panel: integrated CSN (ʃCSN) activity. Arrows: time of intracarotid administration of saline or NaCN. (c) Group data of CSN activity expressed as percentage change from baseline from saline (open circles) and NaCN (filled circles) treated dogs. *n* = 9; in nine dogs saline and NaCN‐mediated effects on CSN were compared during euglycaemia. All data are shown as the mean ± SD. Data were statistically compared by repeated measures two‐way ANOVA (treatment × time) followed by Šidák's multiple comparisons test. *P*‐values for *post hoc* analysis: *P = 0.0220, **P = 0.0098, ****P < 0.001 compared to the corresponding values from the saline treatment. ^†^ *P* < 0.0075 compared to the NaCN baseline values

**FIGURE 2:**
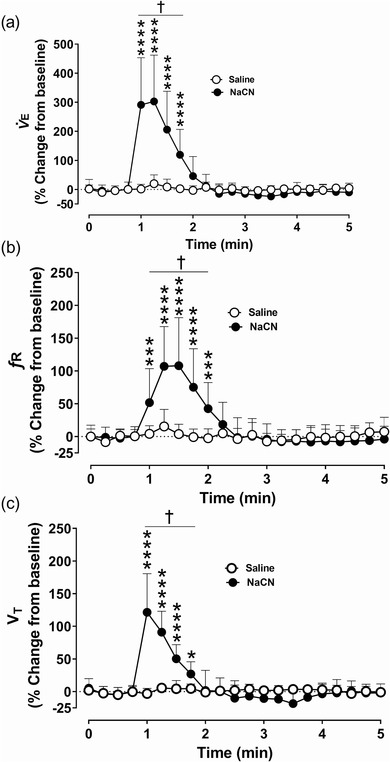
Sodium cyanide (NaCN) transiently increased ventilation during euglycaemia in dogs. Group data showing minute ventilation ($\dot{V}$_E_) (a), respiratory frequency (*f*_R_) (b) and tidal volume (*V*_T_) (c) from euglycaemic spontaneously breathing dogs following intracarotid administration of saline (open circles) and NaCN (filled circles). *n* = 9; in nine dogs saline and NaCN‐mediated effects on ventilation were compared during euglycaemia. All data are shown as the mean ± SD. Data were expressed as percentage change from baseline and statistically compared by repeated measures two‐way ANOVA (treatment × time) followed by Šidák's multiple comparisons test. *P*‐values for *post hoc* analysis: *P = 0.0284, ***P < 0.0003, ****P < 0.0001 compared to the corresponding values from the saline treatment. ^†^ *P* < 0.0123 compared to the NaCN baseline values

Administration of saline into common carotid arteries was without an effect on carotid sinus nerve (CSN) activity (Figure 1a). Compared to saline, administration of sodium cyanide (NaCN; 25 µg/kg) into common carotid arteries elicited an immediate and transient increase in CSN action potentials, and the peak response was significantly larger compared to the baseline and the saline‐treated group (*P* = 0.0001; *n* = 9) during euglycaemia (Figure 1b, c). Both the frequency and the amplitude of compound action potentials increased with NaCN administration (Figure 1c). Consistent with the increase in CSN activity, targeted delivery of NaCN transiently and significantly (*P* = 0.0001) increased the minute ventilation (${\dot{V}}_{E}$) in beagle dogs compared to the baseline and the saline‐treated group (Figure 2a). The increase in ${\dot{V}}_{E}$ was driven by both the respiratory frequency (*f*_R_; *P* = 0.0001, Figure 2b) and the tidal volume (*V*_T_; *P =* 0.0001; Figure 2c). Equal volume of saline administration into both common carotid arteries had no effects on ${\dot{V}}_{E}$, *f*_R_ and *V*_T_ (Figure 2a–c).

**FIGURE 3:**
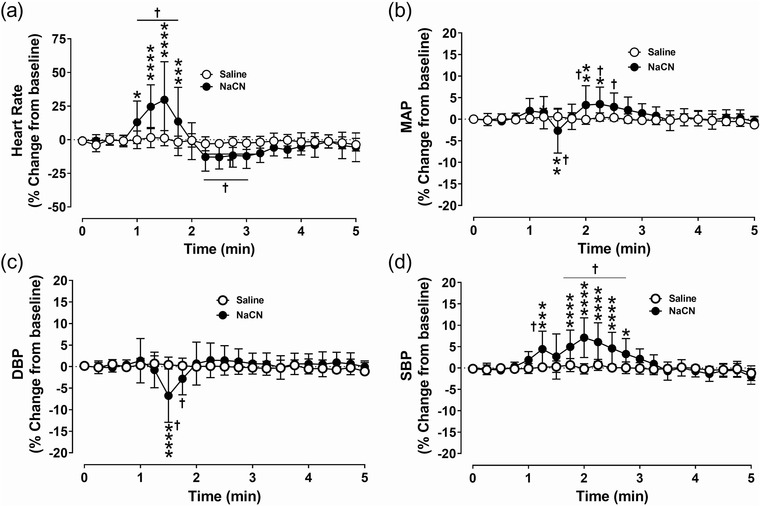
Acute cardiovascular effects of carotid chemoreceptor stimulation during euglycaemia. Group data showing heart rate (a), mean arterial blood pressure (MAP) (b), diastolic blood pressure (DBP) (c) and systolic blood pressure (SBP) (d) from anaesthetized spontaneously breathing dogs following intracarotid administration of saline (open circles) and NaCN (filled circles) during euglycaemia. *n* = 9; in nine dogs saline and NaCN‐mediated effects on heart rate and arterial blood pressure were compared during euglycaemia. All data are shown as the mean ± SD. Data were expressed as percentage change from baseline and were statistically compared by repeated measures two‐way ANOVA (treatment × time) followed by Šidák's multiple comparisons test. *P*‐values for *post hoc* analysis: *P < 0.0155, **P < 0.0071, ***P = 0.0009, ****P < 0.0001 compared to the corresponding values from the saline treatment. ^†^ *P* < 0.0219 compared to the NaCN baseline values

Activation of the carotid chemoreceptors with NaCN transiently increased heart rate (HR; Figure 3a) and changed blood pressure in anaesthetized spontaneously breathing dogs. The HR showed a transient and significant (*P =* 0.0001) increase with NaCN administration (Figure 3a). The mean arterial blood pressure (MAP) showed a biphasic response, initial decrease (*P* = 0.0073) followed by an increase (*P* = 0.0046; Figure 3b). The diastolic blood pressure (DBP) decreased very transiently (*P =* 0.0001) and then recovered to the pre‐NaCN baseline (Figure 3c). The systolic blood pressure (SBP; *P =* 0.0001) significantly increased with NaCN, and the increase lasted for 2–3 min compared to MAP and DBP (Figure 3d). Equal volume of saline administration into both common carotid arteries had no effects on HR, MAP, DBP and SBP (Figure 3a–d).

### 3.2 Effect of insulin on carotid sinus nerve activity and cardiorespiratory parameters

**FIGURE 4:**
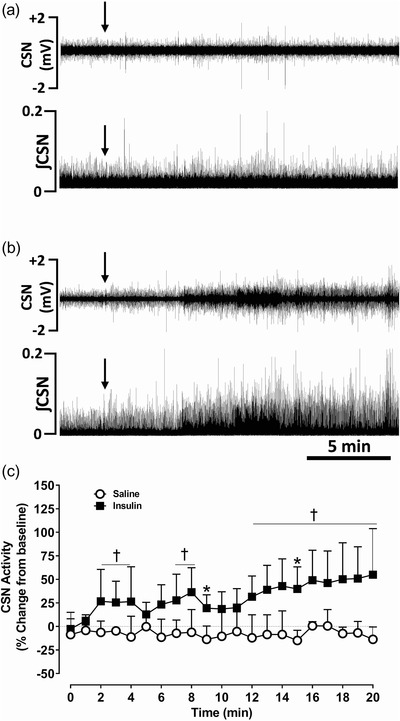
Insulin and insulin‐induced hypoglycaemia had sustained effects on carotid sinus nerve activity in dogs. (a, b) Representative recordings of in vivo carotid sinus nerve (CSN) activity following intracarotid administration of saline (a) and insulin (25 units) (b). Upper panel: CSN raw signal; lower panel: integrated CSN (ʃCSN) activity. Arrows: time of saline or insulin administration. (c) Group data of CSN activity following intracarotid administration of saline (open circles) and insulin (filled squares). *n* = 6; in six dogs, saline and insulin‐mediated effects on CSN were compared. All data are shown as the mean ± SD. Data were expressed as percentage change from baseline and statistically compared by repeated measures two‐way ANOVA (treatment × time) followed by Šidák's multiple comparisons test. *P*‐values for *post hoc* analysis: *P < 0.0491 compared to the corresponding values from the saline treatment. ^†^ *P* < 0.0444 compared to the insulin baseline values

Previous studies have shown that the intravenous infusion (Bin‐Jaliah et al., 2004; Thompson et al., 2016) or intracarotid bolus administration of insulin (Ribeiro et al., 2013) caused CBC‐dependent increase in ${\dot{V}}_{E}$ in rats; however, direct effects of insulin on CSN activity was not known. Therefore, insulin‐mediated effects on CSN neurogram and cardiorespiratory parameters in beagle dogs were studied following administration of insulin into the common carotid artery. Bolus administration of insulin (12.5–25–50–100 µU) into the common carotid artery elicited significant hypoglycaemia (*P =* 0.0001; Table 1) and significant changes in arterial pH (*P =* 0.0457) and $P_{CO_{2}}$ ($P_{\mathrm{aC}O_{2}}$_;_ *P =* 0.0097; Table 1). The end tidal CO_2_ ($ET_{CO_{2}}$; *P =* 0.034) and total CO_2_ (*P =* 0.0134) significantly increased following insulin administration (Table 1). Intracarotid administration of saline had no effects on the CSN neurogram (Figure 4a, c). Insulin significantly increased the CSN action potentials within 2–5 min compared to the pre‐insulin baseline (*P =* 0.0491) and the saline group, and the response reached a maximum within 20 min (*P* = 0.0001; Figure 4b, c). Although the onset of insulin‐mediated increase in CSN action potentials was delayed, the effect was sustained and significant (Figure 4b, c).

**TABLE 1:** Arterial blood gases, pH and glucose values during euglycaemia and insulin‐induced hypoglycaemia

Variables	Euglycaemia	Hypoglycaemia
pH	7.30 ± 0.04	7.24 ± 0.01*
$P_{\mathrm{aC}O_{2}}$ (mmHg)	47.18 ± 5.00	58.22 ± 8.31**
$ET_{CO_{2}}$ (mmHg)	39.69 ± 6.55	48.06 ± 5.73***
Total CO_2_ (mmHg)	24.26 ± 1.63	27.53 ± 1.74****
HCO_3_^−^	22.73 ± 1.50	24.76 ± 2.77
$P_{aO_{2}}$ (mmHg)	100.50 ± 11.39	110.30 ± 10.58
$S_{pO_{2}}$ (mmHg)	95.70 ± 1.9	95.89 ± 2.20
Glucose (mg/dl)	138.90 ± 10.61	45.00 ± 5.38*****

Intracarotid bolus administration of insulin significantly changed arterial blood gases, pH and glucose compared to baseline euglycaemic values. *n* = 6, in six dogs; insulin‐induced hypoglycaemic effects on arterial blood gases, pH and $ET_{CO_{2}}$ were compared. Data are presented as means ± SD. Euglycaemic and insulin‐induced hypoglycaemic data were analysed using an unpaired *t*‐test. *P*‐values for *post hoc* analysis: *P = 0.0457, **P = 0.0097, ***P = 0.0340, ****P = 0.0134, ****P = 0.0001 compared to the euglycaemia values.

**FIGURE 5:**
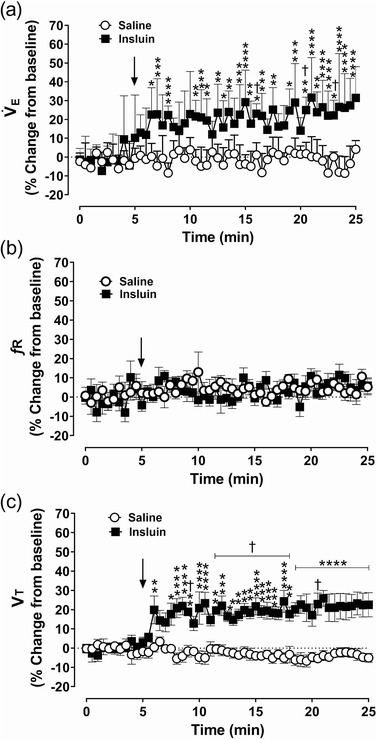
Acute ventilatory effects of insulin and/or insulin‐induced hypoglycaemia in dogs. Group data of minute ventilation ($\dot{V}$_E_) (a), respiratory frequency (*f*_R_) (b) and tidal volume (*V*_T_) (c) from anaesthetized spontaneously breathing dogs following intracarotid administration of saline (open circles) and insulin (filled squares). *n* = 6; in six dogs, saline and insulin‐mediated effects on ventilation were compared. All data are shown as the mean ± SD. Data were expressed as percentage change from baseline and statistically compared by repeated measures two‐way ANOVA (treatment × time) followed by Šidák's multiple comparisons test. *P*‐values for *post hoc* analysis: *P < 0.0444, **P < 0.0096,***P < 0.0007,****P < 0.0001 compared to the corresponding values from the saline treatment. ^†^ *P* < 0.0457 compared to the insulin baseline values

**FIGURE 6:**
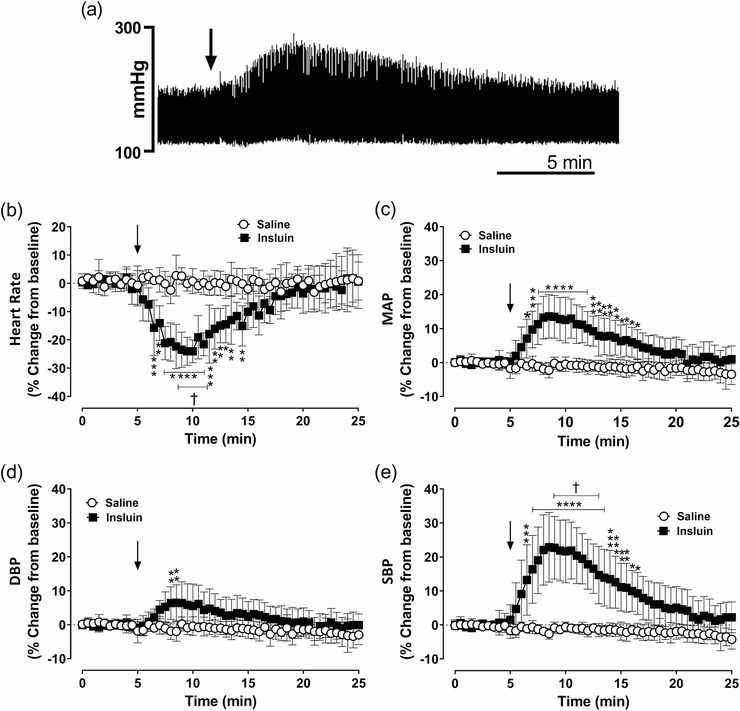
Acute cardiovascular effects of insulin and/or insulin‐induced hypoglycaemia in dogs. (a) Representative recording of arterial blood pressure waveform following intracarotid administration of insulin (25 Units). (b–e) Group data showing heart rate (b), mean arterial blood pressure (MAP) (c), diastolic blood pressure (DBP) (d) and systolic blood pressure (SBP) (e) from spontaneously breathing dogs followed by intracarotid administration of saline (open circles) and insulin (filled squares). *n* = 6; in six dogs, saline and insulin‐mediated effects on heart rate and arterial blood pressure were compared. All data are shown as the mean ± SD. Data were expressed as percentage change from baseline and were statistically compared by repeated measures two‐way ANOVA (treatment × time) followed by Šidák's multiple comparisons test. Arrow: time of saline or insulin administrations. *P*‐values for *post hoc* analysis: *P < 0.0331, **P < 0.0079, ***P < 0.0008, ****P < 0.0001 compared to the corresponding values from the saline treatment. ^†^ *P* < 0.0474 compared to the insulin baseline values

Administration of insulin significantly increased ${\dot{V}}_{E}$ in anaesthetized dogs compared to the pre‐insulin baseline (*P* = 0.0001) and the saline administration (*P =* 0.0078; Figure 5a). Insulin‐mediated increase in ${\dot{V}}_{E}$ was driven by increase in *V*_T_ (*P =* 0.0001; Figure 5c) and not by the change in *f*_R_ (Figure 5b) suggesting that the ventilatory stimulant effects of insulin and those of NaCN are mediated through different mechanisms. Like insulin‐mediated CSN activity, insulin‐mediated onset of ${\dot{V}}_{E}$ was also delayed (Figure 5a–c). Insulin administration produced marked bradycardia and blood pressure changes in anaesthetized dogs (Figure 6a). Insulin administration caused a −24.0 ± 4.0% decrease in HR (*P =* 0.0001; Figure 6b), 13.6 ± 6.4% increase in MAP (*P* = 0.0001; Figure 6c), 6.5 ± 5.3% increase in DBP (*P* = 0.0001; Figure 6d) and 22.9 ± 10.3% increase in SBP (*P =* 0.0001; Figure 6e) compared to the saline‐treated group and the pre‐insulin baseline.

### 3.3 Insulin‐induced hypoglycaemia augmented carotid body chemoreceptor activity and cardiorespiratory responses

**FIGURE 7:**
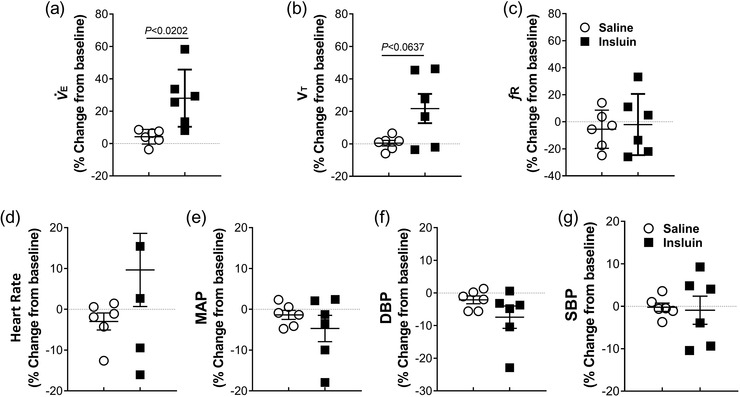
Acute insulin‐induced hypoglycaemia increased baseline ventilation but did not change heart rate and blood pressure in dogs. (a–c) Baseline minute ventilation ($\dot{V}$_E_) (a), tidal volume (*V*_T_) (b) and respiratory frequency (*f*_R_) (c) from spontaneously breathing dogs ∼30 min post‐intracarotid administration of saline (open circles) and insulin (filled squares). (d–g) Baseline heart rate (d), mean arterial blood pressure (MAP) (e), diastolic blood pressure (DBP) (f) and systolic blood pressure (SBP) (g) following intracarotid administration of saline (open circles) and insulin (filled squares). *n* = 6; in six dogs, baseline cardiorespiratory parameters were compared between euglycaemia and insulin‐induced hypoglycaemia (∼30 min post‐insulin administration). All data are shown as the mean ± SD. Data were expressed as percentage change from baseline and were statistically compared by unpaired parametric *t*‐test with Welch's correction

To understand the effects of acute insulin‐induced hypoglycaemia on carotid chemoreceptor sensitivity and cardiorespiratory responses, we compared the NaCN‐mediated CSN neurogram and cardiorespiratory reflex responses during euglycaemia and after insulin‐induced hypoglycaemia. The resting cardiorespiratory parameters were assessed ∼30 min post‐insulin administration and compared to the pre‐insulin values. The resting ${\dot{V}}_{E}$ significantly increased following insulin administration (*P =* 0.0202; Figure 7a), which was driven by increase in *V*_T_ (*P =* 0.0637; Figure 7b) and not by change in *f*_R_ (Figure 7c; *P =* 0.7596). There was no significant change in resting HR (Figure 7d), MAP (Figure 7e), DBP (Figure 7f) and SBP (Figure 7g) ∼30 min into insulin‐induced hypoglycaemia. There was no change in resting cardiorespiratory parameters with saline administration (Figure 7a–g).

**FIGURE 8:**
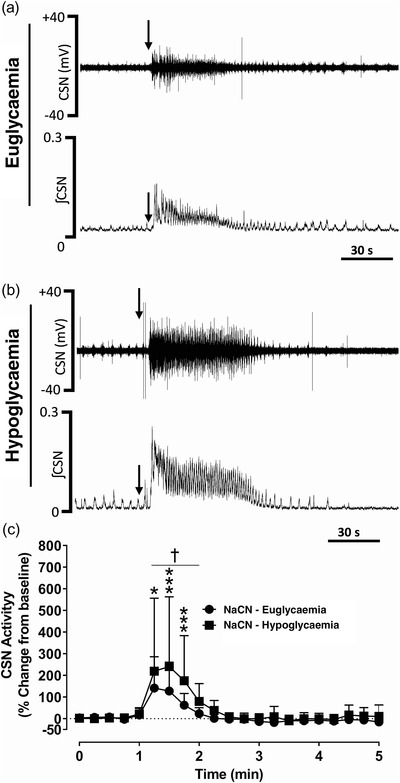
NaCN‐mediated carotid chemoreceptor activity augmented during insulin‐induced hypoglycaemia in dogs. (a, b) Representative in vivo carotid sinus nerve (CSN) recordings following intracarotid administration of NaCN (25µg/kg, arrows) during euglycaemia (a) and insulin‐induced hypoglycaemia (b). Upper panel: CSN raw signal and lower panel: integrated CSN (ʃCSN) activity. Arrows: time of intracarotid NaCN administration. (c) Group data of NaCN‐mediated CSN activity expressed as percentage change from baseline euglycaemia (filled circle) and insulin‐induced hypoglycaemia (filled square). *n* = 6, in six dogs, saline and NaCN‐mediated effects on CSN were compared between euglycaemia and insulin‐induced hypoglycaemia. All data are shown as the mean ± SD. Data were statistically compared by repeated measures two‐way ANOVA (treatment × time) followed by Šidák's multiple comparisons test. *P*‐values for *post hoc* analysis: *P = 0.0309, ***P = 0.001 compared to the corresponding values during euglycaemia. ^†^ *P* < 0.0233 compared to the baseline values

**FIGURE 9:**
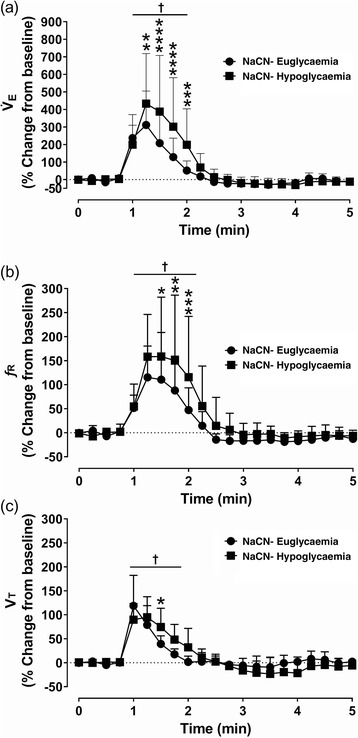
NaCN‐mediated respiratory activity augmented during insulin‐induced hypoglycaemia in dogs. (a–c) Group data of minute ventilation ($\dot{V}$_E_) (a), respiratory frequency (*f*_R_) (b) and tidal volume (*V*_T_) (c) from anaesthetized spontaneously breathing dogs following intracarotid administration of NaCN during euglycaemia (filled circles) and insulin‐induced hypoglycaemia (filled squares). *n* = 6; in six dogs, saline and NaCN‐mediated effects on ventilation were compared between euglycaemia and insulin‐induced hypoglycaemia. All data are shown as the mean ± SD. Data were expressed as percentage change from baseline and statistically compared by repeated measures two‐way ANOVA (treatment × time) followed by Šidák's multiple comparisons test. *P*‐values for *post hoc* analysis: *P < 0.0337, **P < 0.0079, *** *P* < 0.0004, **** P < 0.0001, compared to the corresponding values during euglycaemia. ^†^ *P* < 0.0056 compared to the baseline

**FIGURE 10:**
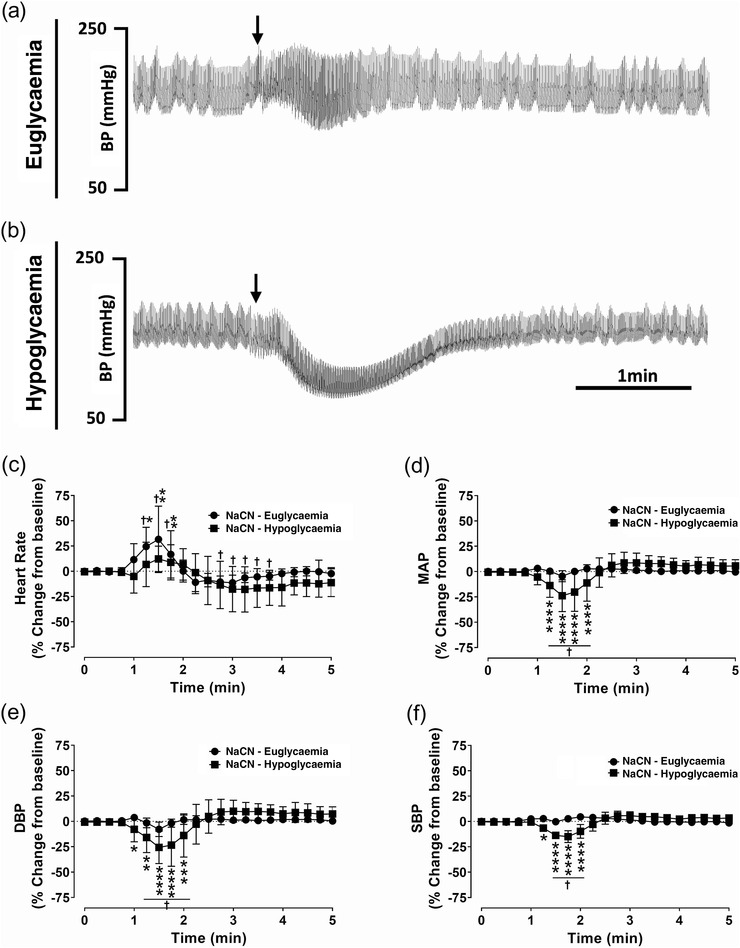
Chemoreceptor stimulation‐mediated blood pressure response augmented during insulin‐induced hypoglycaemia in dogs. (a, b) Representative recordings of arterial blood pressure (BP) waveforms following intracarotid administration of NaCN (25µg/kg, arrows) during euglycaemia (a) and insulin‐induced hypoglycaemia (b). Arrows: time of intracarotid NaCN administration. (c–f) Group data showing heart rate (c), mean arterial blood pressure (MAP) (d), diastolic blood pressure (DBP) (e) and systolic blood pressure (SBP) (f) from anaesthetized spontaneously breathing dogs following intracarotid administration of NaCN during euglycaemia (filled circles) and insulin‐induced hypoglycaemia (filled squares). Data are expressed as percentage change from baseline. *n* = 6; in six dogs, saline and NaCN‐mediated effects on heart rate and arterial blood pressure were compared between euglycaemia and insulin‐induced hypoglycaemia. All data are shown as the mean ± SD. NaCN‐mediated heart rate and blood pressure data obtained during euglycaemia and insulin‐induced hypoglycaemia were analysed using two‐way ANOVA (treatment × time) followed by Šidák's multiple comparisons test. *P*‐values for *post hoc* analysis: *P < 0.0337, **P < 0.006, ***P = 0.0006, ***P < 0.0001 compared to the corresponding values during euglycaemia. ^†^ *P* < 0.0473 compared to the baseline

For the same dose of NaCN (25 µg/kg), the CSN activity was significantly augmented during insulin‐induced hypoglycaemia compared to euglycaemia (*P =* 0.0001; for the maximum peak response; Figure 8a–c). Both amplitude and duration of the CSN action potentials increased during hypoglycaemia (Figure 8b, c). Similarly, NaCN‐mediated ${\dot{V}}_{E}$ was also significantly larger during hypoglycaemia compared to euglycaemia (*P =* 0.0001, Figure 9a) and this increase in ${\dot{V}}_{E}$ was mostly driven by *f*_R_ (*P =* 0.0002; Figure 9b) and not due to a change in *V*_T_ (Figure 9c). The CBC stimulation‐mediated change in cardiovascular reflex response was significantly larger during hypoglycaemia in anaesthetized dogs (Figure 10a, b). CBC stimulation during hypoglycaemia resulted in a larger and significant decrease in MAP (*P =* 0.0001; Figure 10d), DBP (*P =* 0.0001; Figure 10e) and SBP (*P =* 0.0001; Figure 10f) with attenuated HR responses compared to euglycaemia. The CBC stimulation‐mediated change in HR was significantly attenuated during hypoglycaemia compared to euglycaemia (*P =* 0.0061, Figure 10b) suggesting despite the larger and significant decrease in blood pressure with activation of CBC, the typical baroreflex‐mediated rise in HR was blunted in dogs during hypoglycaemia (Figure 10c).

## 4 DISCUSSION

The present study demonstrated for the first time direct effects of insulin and/or insulin‐induced hypoglycaemia on CBC activity and established that an altered glycaemic state changes the counter‐regulatory reflex responsiveness of the peripheral chemoreceptors, baroreceptors and cardiorespiratory system. During insulin‐induced hypoglycaemia, stimulation of the CBCs with NaCN leads to augmented CSN activity, ${\dot{V}}_{E}$ and blood pressure compared to euglycaemia, suggesting enhanced sensitivity of the peripheral chemoreceptors and/or baroreceptors through counter‐regulatory pathways. In addition to the direct effects on the central nervous system, intracarotid administration of insulin significantly increased the CSN activity and ${\dot{V}}_{E}$ with profound effects on HR and blood pressure, suggesting a strong modulatory effects of insulin on peripheral chemoreceptors and/or baroreceptor afferents that control the cardiorespiratory neuronal networks in the brainstem. Despite the exacerbated cardiorespiratory reflex responses, the baroreflex response mediated through chemoreceptor activation was significantly blunted during insulin‐induced hypoglycaemia.

### 4.1 NaCN, a potent carotid chemoreceptor and cardiorespiratory stimulant

NaCN has been extensively used as a selective CBC stimulant across different animal models (for review see Marshall, 1994). In dogs, NaCN has been used as a pharmacological tool to selectively stimulate the CBCs (Alvarez‐Buylla & de Alvarez‐Buylla, 1988, 1994; Tsuchiya et al., 1990) or to validate the physiological viability of the CBCs after anatomical interventions (Koyama et al., 2000; Rodman et al., 2001; Pijacka et al., 2018). However, very few studies have reported NaCN‐mediated changes in CSN activity in dogs (Alvarez‐Buylla & de Alvarez‐Buylla, 1988). In the present study, NaCN produced an immediate onset of response on the CSN neurogram with intracarotid administration, which further corroborates previous findings in rats (Bisgard et al., 2003), rabbits (Matsumoto, 1986), cats (Iturriaga & Alcayaga, 2007; Mulligan et al., 1981) and dogs (Alvarez‐Buylla & de Alvarez‐Buylla, 1988). Furthermore, NaCN significantly increased ${\dot{V}}_{E}$ (>200% increase) in dogs, which was driven by both *f*_R_ and *V*_T_. A similar ventilatory responses to NaCN was also reported in rats (Sugito et al., 1998), rabbits (Matsumoto, 1986), cats (Berger, 1980), dogs (Alvarez‐Buylla & de Alvarez‐Buylla, 1988) and goat (Pan et al., 1998). It has been shown that NaCN‐dependent increase in ${\dot{V}}_{E}$ was mediated by the CBCs, as bilateral denervation of CSN (CSNx) attenuated the NaCN‐mediated ventilatory response (Koyama et al., 2000; Rodman et al., 2001).

Similarly, stimulation of peripheral chemoreceptors in spontaneously breathing dogs resulted in a significant and transient change in HR, SBP and DBP. The cardiovascular responses to selective stimulation of the CBCs depends on an interplay between the primary response of CBC excitation, secondary reflex mechanisms of pulmonary stretch receptors and arterial baroreceptors, and finally the direct actions of stimuli on vasculature, heart and the central nervous system (Marshall, 1994). For example, stimulation of the CBCs resulted in bradycardia when the change in ventilation was <200% and tachycardia when the ventilation was >200% (Daly & Scott, 1963; Marshall, 1994). The tachycardic response that we observed in the present study further confirms the magnitude of the NaCN‐mediated increase in ${\dot{V}}_{E}$ (Figure 2a). Further, stimulation of CBCs following denervation of the lung‐innervating nerves changes the tachycardia into bradycardia, suggesting the importance of lung stretch receptors in determining HR response secondary to hyperventilation in dogs, and the pulmonary stretch receptor reflex decreases vascular resistance (Daly & Scott, 1963) and increases HR via central vagal inhibition in response to increased *V*_T_ (Paintal, 1973). The biphasic response in MAP following CBC stimulation corresponds with the MAP response reported in dog (Rutherford & Vatner, 1978) and humans (Tubek et al., 2016) following by CBC stimulation with gaseous hypoxia and pharmacological agents. The first phase of MAP response may be associated with the primary CBC‐evoked sympathoexcitation (Xie et al., 2001) and the secondary phase may be attributed to activation of pulmonary stretch receptors (Tubek et al., 2016). Furthermore, hyperventilation evoked by CBC stimulation results in hypocapnia, which induces tachycardia and peripheral vasodilatation (Marshall, 1994). The vasodilator effects of hypocapnia may be attributed to its inhibition of sympathetic vasoconstrictor activity produced by the influence of hypocapnia on the neuronal population along the ventromedial medulla and the interior mediolateral column of the spinal cord (Guyenet, 2000).

### 4.2 Insulin, a peripheral chemoreceptor modulator

Although several *ex vivo* studies have indicated that insulin can stimulate the CBCs, we for the first time demonstrated that in vivo intracarotid administration of insulin increased the CSN neurogram in the dog. Previously, incubation of isolated rat whole carotid body preparation in high insulin‐containing medium increased [Ca^2+^]_i_ and elicited the release of ATP and dopamine in a dose‐dependent manner (Ribeiro et al., 2013) suggesting direct action of insulin on the CBCs. This has been further supported by the presence of insulin receptors and their phosphorylation in response to insulin treatment in the rat carotid body (Ribeiro et al., 2013). Further, acute bilateral CSNx attenuated insulin‐mediated increase in ${\dot{V}}_{E}$ (Ribeiro et al., 2013), suggesting the CBCs are the primary site of action for stimulating the neurosecretory pathway to drive CSN activity, sympathoexcitation and cardiorespiratory reflex responses. A direct action of insulin on central chemoreceptors cannot be ruled out because insulin crosses the blood–brain barrier, and its receptors are also reported in the respiratory centres in the brainstem. Administration of insulin increased ${\dot{V}}_{E}$ in dogs, which was driven by increase in *V*_T_, not by the change in *f*_R_. The time course for the onset of insulin‐mediated effects on CSN and respiratory parameters was different from that of NaCN (Figure 5). Intracarotid administration of NaCN produced an immediate and transient increase in ${\dot{V}}_{E}$ whereas insulin‐mediated increase in ${\dot{V}}_{E}$ was delayed (2–5 min) but sustained, reaching maximum response at 15–20 min, suggesting two independent mechanisms to drive ventilation in dog. A similar time profile for insulin‐mediated increase in ${\dot{V}}_{E}$ was observed in rodents with intracarotid administration, which is in accordance with the time scale necessary for the activation of tyrosine kinase receptors, namely, the insulin receptors (Ribeiro et al., 2013; Melo et al., 2022). On the other hand, NaCN acts by inhibiting cytochrome oxidase in the type‐1 glomus cells in the carotid body to elicit the ventilatory response (Wang et al., 2017). Interestingly, independent of the glycaemic condition, insulin‐mediated increase in ${\dot{V}}_{E}$ was reported in rats (Bin‐Jaliah et al., 2004; Ribeiro et al., 2013; Thompson et al., 2016) and humans (Limberg et al., 2014; Barbosa et al., 2018) suggesting an independent effect of insulin on ${\dot{V}}_{E}$ where the increase was driven by both *f*_R_ and *V*_T_.

Insulin administration produced a significant increase in blood pressure (DBP, SBP and MAP) with a sharp decrease in HR, which started within a few minutes post‐insulin dose with the peak response reached at 8–10 min. The early onset of the insulin‐mediated pressor response may be due to active vasoconstriction, not as a result of the direct action of insulin on the blood vessels; rather these changes appeared to be mediated by the activation of the sympathetic system which originates in the CBC–central nervous system (arcuate–paraventricular nucleus to nucleus tractus solitarus) axis. Hyperinsulinaemia‐mediated cardiovascular responses were reported within 20–45 min post‐intravenous administration of insulin, when the blood sugar level reached a critical low point (40–51 mg/dl), suggesting a true hypoglycaemic stimulation of the hypothalamus–brainstem with excitation of the sympathetic nerves and release of catecholamines (Pereda et al., 1962). Moreover, intracarotid insulin resulted in higher blood pressure and sympathetic activity compared to systemic insulin administration. Therefore, based on the time profile of insulin‐mediated change in blood pressure and HR, it is possible that insulin has direct effects on the CBC–central nervous system axis to trigger the cardiorespiratory responses. This corroborates the acute change in pressor response and cardiac output with the intracarotid administration of insulin in dogs (Pereda et al., 1962).

Functionally, insulin has opposing effects on haemodynamics as vasodilator and vasoconstrictor (Muniyappa et al., 2007). Even various parts of the arterial vascular network (distal and proximal arterioles) have a differential response to insulin and sympathetic nervous system. With elevated sympathetic nerve activity, distal arterioles vasodilate in response to insulin, but proximal arterioles undergo sustained vasoconstriction (Marshall, 1994). Studies have shown that in humans, insulin infusion at physiological concentrations stimulates vasodilatation and increased blood flow by regulating endothelial production of the potent vasodilator nitric oxide, by activating phosphatidylinositol 3‐kinase signalling pathways (Muniyappa et al., 2007). Conversely, insulin produces vasoconstriction effects by activating the sympathetic nervous system for the secretion of the vasoconstrictor endothelin‐1 from the vascular endothelium (Scherrer & Sartori, 1997) through distinct mitogen‐activated protein kinase‐dependent insulin‐signalling pathways. In healthy lean individuals, physiological concentrations of insulin increase venous catecholamine levels and sympathetic nerve activity. Insulin also drives sympathoexcitation through its action on the arcuate and paraventricular nucleus in the central nervous system, a carotid body‐independent pathway to activate the sympathetic nervous system (Conde et al., 2018).

### 4.3 Insulin‐induced hypoglycaemia elevated the resting ventilation

Insulin‐induced hypoglycaemia significantly elevated resting ${\dot{V}}_{E}$ (30% compared to euglycaemia), predominantly by an increase in *V*_T_. Hypoglycaemia‐mediated increase in ${\dot{V}}_{E}$ was consistent with previous studies in rats (∼25% increase; Bin‐Jaliah et al., 2004; Ribeiro et al., 2013; Thompson et al., 2016) and humans (∼54% increase; Ward et al., 2007; Limberg et al., 2014). The elevated resting ${\dot{V}}_{E}$ during hypoglycaemia precisely matches the concurrent rise in metabolic rate, thereby preventing metabolic alkalosis and rise in $P_{\mathrm{aC}O_{2}}$(Bin‐Jaliah et al., 2004; Thompson et al., 2016). Therefore, the elevated baseline $\dot{V}$_E_ during the insulin induced‐hypoglycaemia is the result of a highly sensitive counter‐regulatory response to activate the autonomic nervous system to normalize the elevated metabolic rate and altered arterial blood gases. Despite the increased resting ${\dot{V}}_{E}$ during hypoglycaemia, the $P_{\mathrm{aC}O_{2}}$, $ET_{CO_{2}}$ and total CO_2_ were significantly elevated, and pH was significantly low (Table 1), suggesting a clear sign of ventilation–perfusion mismatch in the lungs (Table 1). The supplemental oxygen provided to keep the peripheral chemoreceptor quiescent maintained a comparable $P_{aO_{2}}$ level during euglycaemia and hypoglycaemia. Apart from prolonged anaesthesia, the strong vasoconstrictor effects of insulin mediated through activation of the sympathetic nervous system might have contributed for the ventilation–perfusion mismatch that resulted in elevated $P_{\mathrm{aC}O_{2}}$, $ET_{CO_{2}}$, total CO_2_ and low pH (Muniyappa et al., 2007; Ribeiro et al., 2013).

The resting HR and blood pressure did not change significantly during insulin‐induced hypoglycaemia (Figure 7). In rodents, hypoglycaemia had no significant effect on HR and blood pressure after sham and bilateral CSNx (Bin‐Jaliah et al., 2004), suggesting that the contribution of peripheral chemoreceptors/baroreceptors to cardiovascular response during hypoglycaemia is minimal. However, in humans, insulin‐induced hypoglycaemia produced tachycardia and a rise in blood pressure suggesting a strong hyperactivity of the sympathetic nervous system (Fagius, 2003), and this effect in human is mediated by the CBCs. Suppression of CBC activity with either systemic hyperoxia or bilateral CSNx blunts the counter‐regulatory hormonal responses to hypoglycaemia.

### 4.4 Insulin‐induced hypoglycaemia augmented carotid body chemosensitivity and respiratory responses

In dogs, NaCN‐mediated CSN activity and cardiorespiratory responses were significantly augmented during insulin‐induced hypoglycaemia. Consistent with these findings, both hypoxic (Ward et al., 2007) and hypercapnic (Thompson et al., 2016) ventilatory responses were also significantly augmented during hypoglycaemia in human and rodents, respectively. A normal tonic input from the peripheral chemoreceptors to the central chemoreceptors has been shown to be vital and involved in regulation of various cardiorespiratory functions in normal physiology (Kumar & Prabhakar, 2012). However, CBC hyperactivity as reported in various pathophysiological conditions, such as hypertension, heart failure, sleep apnoea and more recently in metabolic disorders (Paton et al., 2013), engages the sympatho‐adrenal axis‐mediated sympathetic outflow, which in turn exacerbates the reflex responsiveness of the CBCs and the cardiorespiratory systems.

Mechanistically, apart from hyperinsulinaemia, hypoglycaemia‐mediated counter‐regulatory adrenaline and the β‐adrenoreceptor pathway contributed to elevated CBC activity because either blockade of β‐adrenoreceptor with propranolol or adrenalectomy abolished the hypoglycaemia‐mediated increase in ${\dot{V}}_{E}$ (Thompson et al., 2016). In fact, studies have shown two‐ to three‐fold increase in plasma noradrenaline and over 10‐fold increase in adrenaline during insulin‐induced hypoglycaemia in healthy humans (Tanaka et al., 2022; for references see Fagius, 2003). Exogenous adrenaline, noradrenaline or β‐adrenoreceptor agonists augments ventilation in multiple species, an effect that is dependent on the CBCs (Bin‐Jaliah et al., 2005; Thompson et al., 2016). Apart from hypoglycaemia‐mediated counter‐regulatory hormones, factors such as augmented metabolism or proinflammatory cytokines may enhance the sensitivity of the peripheral chemoreceptors during hypoglycaemia.

### 4.5 Insulin induced hypoglycaemia exacerbated cardiovascular reflex responses

Activation of the CBCs with NaCN during hypoglycaemia exacerbated the arterial blood pressure response compared to euglycaemia with attenuated cardiac baroreflex response to increase HR. We speculate that the hyperactive sympathetic nervous system is responsible for the exaggerated blood pressure response during hypoglycaemia. The cardiovascular reflex responses to CBC activation in spontaneously breathing dog results in tachycardia, an increase in cardiac output, fall in systemic blood pressure and decrease in total peripheral vascular resistance (Daly & Scott, 1963). Similar to humans (Limberg et al., 2014), our study also provides evidence for the pivotal role of the CBCs in the regulation of integrative physiology including blood glucose homeostasis and baroreflex control of blood pressure in dogs. In normal conditions, a transient fall in blood pressure initiates the baroreceptor reflex response to increase HR, contractility of the heart, vascular resistance and venous return to maintain blood pressure at an optimum level (Marshall, 1994). This ability to adapt to challenging conditions is known as baroreflex sensitivity. Lack of baroreflex sensitivity to increased HR during a transient fall in blood pressure suggests a blunted baroreflex response. In humans, activation of the peripheral chemoreceptors with hypoxia (Cooper et al., 2005) and hypoglycaemia (Limberg et al., 2014) or desensitizing/inhibiting the CBCs with systemic hyperoxia (Wehrwein et al., 2010; Wehrwein et al., 2015) decreases sympathetic baroreflex sensitivity, suggesting baroreflex control of the blood pressure may be altered by activation or inhibition of the CBCs. Therefore, the exacerbated cardiovascular responses along with blunted baroreflex response during hypoglycaemia suggest a significant role of glycaemic state in maintaining HR and arterial blood pressure.

In conclusion, this study demonstrates for the first time that independent of blood glucose levels, insulin and/or insulin‐induced hypoglycaemia modulates the peripheral chemoreceptors by increasing the afferent action potentials of CSN and increasing the ${\dot{V}}_{E}$ with significant cardiovascular responses in anaesthetized beagle dogs. Similarly, augmented chemoreceptor and cardiorespiratory responses with peripheral chemoreceptor and/or baroreceptor stimulation during insulin induced‐hypoglycaemia suggests that hypoglycaemia‐mediated release of counter‐regulatory hormones including catecholamines, adrenaline and noradrenaline augmented the peripheral chemoreceptor sensitivity. Although insulin is known to have independent effects on sympathoexcitation and peripheral vasodilatation, the marked cardiovascular and respiratory responses following intracarotid administration of insulin suggest overactive sympathetic outflow to enhance the sensitivity of the CBC. Therefore, controlling the overactive sympathetic outflow to the CBCs by pharmacological agents and/or by bioelectronic modulation might be beneficial for various pathophysiological conditions, such as hypertension, heart failure and sleep apnoea.

## AUTHORS CONTRIBUTION

All animal experiments were performed at the GSK, Upper Merion, King of Prussia. Santhosh M. Baby, Robert W. Coatney, Faisal Zaidi, Gerald E. Hunsberger, David Sokal, and Daniel Chew designed the experiments. Santhosh M. Baby, Faisal Zaidi, Gerald E. Hunsberger, and Robert W. Coatney performed the experiments. Santhosh M. Baby, Gerald E. Hunsberger, Isha Gupta, and Robert W. Coatney analyzed the data and wrote the manuscript. Silvia V. Conde, Kristen Rall, David Sokal, and Daniel Chew actively participated in the interpretation of the results. All authors have approved the final version of the manuscript and agree to accountable for all aspects of the work. All persons designated as authors qualify for authorship, and all those qualify for authorship are listed.

## CONFLICT OF INTEREST

Santhosh M. Baby, Faisal Zaidi, Gerald E. Hunsberger, David Sokal, Isha Gupta, Kristen Rall, Daniel Chew and Robert W. Coatney are employed by Galvani Bioelectronics. Silvia V. Conde declares that Galvani Bioelectronics provided funds to support their work associated with Type 2 diabetic project.

## Supporting information

Statistical Summary Document

## Data Availability

The data that support the findings of this study will be made available by the authors upon reasonable request.
